# Prevalence of hookworm infection and associated factors among pregnant women attending antenatal care at governmental health centers in DEMBECHA district, north West Ethiopia, 2017

**DOI:** 10.1186/s12884-020-03134-0

**Published:** 2020-08-12

**Authors:** Sewnet Wongiel Misikir, Moges Wobie, Mengistie Kassahun Tariku, Simachew Animen Bante

**Affiliations:** 1 Department of Medical Laboratory Technologist at Felege Hiote Specialized referral hospital, Bahir Dar, Ethiopia; 2 Department of Public Health, College of Health Sciences, Debre Markos University, Debre Markos, Ethiopia; 3 Resident of Field Epidemiology, Department of Epidemiology and Biostatistics, College of Medicine and Health Sciences, Bahir Dar University, Bahir Dar, Ethiopia; 4 Department of Midwifery, College of Medicine and School of Health Sciences, Bahir Dar, University, Bahir Dar, Ethiopia

**Keywords:** Hookworm, Prevalence, Pregnancy, Antenatal care, Dembecha District, Ethiopia

## Abstract

**Background:**

Hookworm infection is a major public health problem in developing countries. The main way people become infected with hookworm’s larva is through direct skin contact with contaminated soil when walking on barefoot. It is one of a major cause of anemia in pregnant women. The objective was to assess the prevalence and associated factors of hookworm infection among pregnant women who attended antenatal care at governmental health centers in Dembecha district, Ethiopia, 2017.

**Methods:**

Institutional based cross sectional study was conducted on 306 pregnant women. Study participants were selected by systematic random sampling technique from February 1 to March 30, 2017. Data collectors and supervisors were trained. Semi-structured Amharic version questionnaire was used to collect data using face to face interview technique and stool sample was examined. Collected data were entered by using Epi data version 3.1 and exported to SPSS. The exported data was analyzed and presented by using descriptive summary statistics and tables. After bivariate logistic regression analysis, all variables with a *p*-value < 0.25 were entered into multivariate logistic regression and *p* value < 0.05 considered as significantly associated with the outcome variable.

**Results:**

Prevalence of hookworm infection was 32.0%. There was a positive association of hookworm infection with living in single room [AOR =2.8; 95% CI; 1.32–5.81], living with domestic animals [AOR = 3.4; 95% CI; 1.35–8.76], monthly income ≤1500 Ethiopian birr [AOR = 3.7; 95% CI; 1.76–7.64], unavailability of latrine [AOR = 2.2; 95% CI; 1.03–4.55], habit of walking on barefoot [AOR = 4.3; 95% CI; 2.17–8.48] and not habit of hand washing before meal [AOR = 3.4; 95 CI; 1.14–10.12].

**Conclusion:**

This study showed high prevalence of hookworm infection among pregnant women in the study area. Living in single room, living with domestic animals, monthly income ≤1500 Ethiopian birr, unavailability of latrine, habit of walking on barefoot and not habit of hand washing before meal had positive association with hookworm infection. Public health measure should focus on availability latrine and separation of humans and domestic animals room to decrease prevalence of hookworm infection among pregnant women.

## Background

Globally, 700 million people including 44 million pregnant women are infected with hookworm. It is also a cause of 3000 to 65,000 deaths annually [1]. In Sub-Saharan African, 37.7 million women of reproductive age are infected with it and those approximately 6.9 million are pregnant women [2]. In this region, countries with the highest prevalence of hookworm are Nigeria, Democratic Republic of Congo, Angola, Côte d’Ivoire and Ethiopia. It ranks at the top of major causes of disease burden because of the anemia that result from the blood feedings of the adult parasite [3]. The effect of hookworm infection on pregnant women and their fetus greatly varies from asymptomatic to sever infection that results in malnutrition, anemia, intrauterine growth retardation and spontaneous abortion [4]. It is also a leading cause of maternal and child illness in developing countries of the tropics and subtropics [3]. All pregnant women who were infected with hookworm were found to be anemic [5]. Anemia due to hookworm infection costs $20.9 billion worldwide. It also resulted in $11.0 billion in productivity losses in Africa [6]. Majority of infected individuals live in poverty-stricken areas with poor environmental sanitation [1]. The study done in southern region of Ethiopia showed that hookworm infection is the second most prevalent intestinal parasite among pregnant women with the mean prevalent of 7% [7]. Even if there is current global control efforts based on periodic mass anthelmintic administration, it is unsustainable [8]. In Ethiopia, deworming is a component of antenatal care (ANC) but use of drugs to control intestinal parasites during pregnancy is least common among rural women, women with no education and women in households in the lowest and middle wealth quartile. There is limited information about hookworm infection in the study area. Thus, this study aimed at describing the prevalence of hookworm infection among pregnant women attending antenatal care in Dembecha district and determining factors associated with hookworm infection.

## Methods

### Study area and period

Institutional based cross sectional study was conducted from February1 to March 30, 2017 in Dembecha district, Northwest Ethiopia. Dembecha district is bordered by Debre Elias district in the South, Jabitenan district in West, Dega Damot district in the North and Machakle district in the East. It is also located 350 and 217 km away from the capital city of Ethiopia (Addis Ababa) and capital city of Amhara Region (Bihar Dar), respectively. The sea level elevation of the district is 2083 m. Humidity of the district is 54%. The total population of the district was 156,665 and populations’ density is 133.08. The district has a latitude and longitude of 10^o^33’N37^o^29’E/ 10.550^o^N37.483°E with an elevation of 2083 m above sea level. The district has six governmental health centers [9].

### Sample size and sampling technique

The required sample size was computed by using single population proportion formula with the assumption of 95% CI and 7% prevalence rate of hookworm infection among pregnant women which was done in Hossana, Southern Ethiopia [7]. 10% non- response rate and 3% margin error used to obtain total sample size 306. The total sample size was proportionally allocated for the six health centers based on the number of pregnant women who were registered in the health centers. Study participants in each health center were selected by systematic random sampling during the study period. Total number of pregnant women who expected to visit the health centers during data collection period was 881. Sampling fraction (K) was obtained by dividing the total pregnant women who were expected to visit the health centers for the total sample size. The first study participant was selected by lottery method and then every K interval (3rd) interval in each health center was included in the study.

### Data collection procedure

Semi structured questionnaire was used to collect data using face to face interview technique by 6 trained laboratory technicians. The questionnaire had three sections that include socio-demographic information, personal, and environmental characteristics.

Questionnaire was prepared in English and translated into Amharic and translated back into English to check its consistency. The Amharic version questionnaire was used for data collection. Unique code was given for each questionnaire and laboratory request format. After asking the participants consent, they were interviewed and requested to give stool sample in clean dry cup. Similar codes were written on the cups with questionnaire and the laboratory request format. A fecal sample of all study participants was examined using wet mount preparation. A drop of fresh physiological saline was placed on a clean slide approximately 1 g of stool sample was added.

The preparation was covered with cover slip and examined under microscope for the presence or absence of hookworm ova.

### Data quality control

Six diploma laboratory technicians were selected for data collection. And three laboratory professionals were selected for supervision. Training was given for both data collectors and supervisors for 2 days about the objective, process of data collection and standard operating procedure. Pretest was done on 16 pregnant women at Amanual health center in Machakle district before the actual data collection. Some clarifications and correction on the questionnaire was made after the pretest. Standard operating procedure (SOP) was used for laboratory procedures. Close supervision was under taken by supervisors during data collection. Specimens were selected randomly and re-examined by supervisors for cross checking the accuracy of laboratory results.

### Data analysis

Collected data was coded and entered by using Epi data version 3.1 and then it was exported to SPSS version 20 for analysis. Descriptive, bivariate and multivariate analysis was done. After bivariate logistic regression analysis, all variables with a *p*-value less than 0.25 were entered into multivariate logistic regression to identify significant factors for the occurrence of hookworm infection among pregnant women. *P*-value less than 0.05 were used as cut off point for presence of statistical significance. Model goodness of fitness was checked by Hosmer and Lemeshow Test (*p*-value =0.071). Tables and texts were used to present the result of the analyzed data.

## Results

### Socio-demographic characteristics

A total of 306 pregnant women were included in the study with 98.04% response rate. The mean age of the study participants was 26.56 ± 5.94 year. Among the total study participants, 290 (96.7%), 176(58.7%), 286 (95.3%) and165 (55%) were married, rural residence, Orthodox and farmers respectively. Half, 152 (50.7%) of women could not able to read and write. Regarding to monthly income, 208 (69.3%) of the study participants earned > 1500 Ethiopian birr (Table 1).

**Table 1:** Socio-demographic and pregnancy related characteristics of pregnant women who attended antenatal care at governmental health centers in Dembecha district, North West Ethiopia, 2017 (*n* = 300)

Variable	Frequency	Percentage
**Residence**		
Urban	124	41.3
Rural	176	58.7
**Age**		
***≤***18 year	12	4.0
19–24 year	143	47.7
***≥***25 year	145	48.3
**Marital status**		
Married	290	96.7
Single	5	2.0
Divorced	4	1.3
Widowed	1	0.3
**Educational status**		
Unable read and write	152	50.5
Able to read and write but no formal education	33	11
Primary	70	23.3
Secondary	36	12
Above secondary	9	3
**Religion**		
Orthodox	286	95.3
Muslim	10	3.3
Catholic	1	0.3
Protestant	3	1
**Occupation**		
House wife	36	12
Government employee	18	6.0
Farmer	165	55.0
Merchant	73	24.3
Others^a^	8	2.7
**Income**		
> 1500	208	69.3
≤1500	92	30.7
**First pregnancy**		
Yes	127	42.3
No	173	57.7
**If not, number of pregnancy including the present one**		
2	53	30.6
≥ 3	120	69.4
**Month of current pregnancy**		
**≤** 3	84	28
4–6	123	41
≥ 7	93	31

Others^a^ = Daily labors, 6 (2%) and Students, 2 (o.7%).

### Environmental and personal hygiene characteristics

Nearly three-fourth, 211(70.3%) of the respondents were living in house having two or more rooms. More than half, 162 (54%) of the participants were living in households that have toilet facility but majority, 111 (68.5) of participants’ toilets were made in a traditional way. Among participants, 138 (46%) were living in the households without toilet facility, and most 118(85.6%) of them had habit of open defecation. Majority, 202(67.3%) of the respondents have lived with domestic animals. Two hundred nineteen (73%) of participants had habit of taking bath. Most 208(69.3%) of the respondents had the habit of eating raw fruit or vegetable. Less than half 138 (40%) of the participants had habit of walking on barefoot (Table 2).

**Table 2:** Environmental and personal hygiene characteristics among pregnant women who attended at governmental health centers in Dembecha district, North West Ethiopia, 2017 (*n* = 300)

Variables	Frequency	Percentage
**Number of living rooms**		
1	89	29.7
≥ 2	211	70.3
**Availability of latrine**		
Yes	162	54.0
No	138	46.0
**types of latrine(*n*** **= 162)**		
Improved	51	31.5
Traditional	111	68.5
**Use of the latrine(*n*** **= 162)**		
Always	110	67.9
Some times	52	32.1
**Place of defecation(*n*** **= 138)**		
Open field	118	85.6
From neighbor toilet	14	10.1
Others	6	4.3
Open defecation seen		
Yes	159	53
No	141	47
**Location of open defecation seen (*n*** **= 159)**		
Near the river	54	18
In the main road	39	13
Through the bush	137	45.7
Other	5	1.7
**Domestic animal presence**		
Yes	202	67.3
No	98	32.7
**Type of animals**		
Cat	88	29.3
Dog	87	29
Cow/ox	152	50.7
Goat	40	13.3
Sheep	99	33
Others	12	4
**Source of drinking water**		
Pipe	111	37
River	20	6.7
Well	105	35
Spring	64	21.3
**Habit of hand washing before meal**		
Yes	242	80.7
No	58	19.3
**Have you a habit taking of bath**		
Yes	219	73
No	81	27
**How often do you take(*n*** **= 219)**		
**>** 1 a week	43	19.6
Once a week	74	33.8
Once a month	99	45.2
Others	3	1.4
**Where do you take (*n*** **= 219)**		
Inside the house	97	44.3
Outside the house	122	55.7
**Place of bath (*n*** **= 122)**		
Pond	61	50
At water fall	48	39.3
^a^Others	13	10.7
**Habit of raw fruit eating**		
Yes	208	69.3
No	92	30.7
**Washing fruit before eating (*n*** **= 208)**		
Yes	83	39.9
No	125	60.1
**Habit of shoe wearing**		
Yes	162	54
No	138	46

Others^a^ = under the tree, 5 (4.1%), Latrine, 4 (3.3%) and Backyard, 4 (3.3%).

### Prevalence of hookworm infection

Based on stool microscopic result, 96 (32%) 95% CI (26.3–37%) of the respondent had hookworm infection.

### Factors associated with hookworm infection

In Multivariable analysis; monthly income (AOR = 3.7; 95% CI; 1.76–7.64), living with a single room (AOR =2.8; 95% CI; 1.32–5.81), unavailability of latrine (AOR = 2.2; 95% CI; 1.03–4.55), living with domestic animals (AOR = 3.4; 95% CI; 1.35–8.76), habit of walking on barefoot (AOR = 4.3; 95% CI; 2.17–8.48) and not habit of hand washing before meal (AOR = 3.4; 95 CI; 1.14–10.12) were significantly associated with hookworm infection (Table 3).

**Table 3:** Multivariate logistic regression analysis of factors associated with hookworm infection among pregnant women who attended antenatal care at governmental health centers in Dembecha district, North West Ethiopia, 2017 (*n* = 300)

Characteristics	Hookworm infection		COR ((95% CI)	AOR (95% CI)	***P***-value
	No	Yes			
Income					
> 1500	160	48	1	1	
≤ 1500	44	48	3.6 (2.16–6.12)	3.7 (1.76–7.64)	0.001*
Number of living room					
**≥ 2**	155	56	1	1	
1	49	40	2.3 (1.34–3.79)	2.8 (1.32–5.80)	0.007*
Availability of latrine					
Yes	132	30	1	1	
No	72	66	4.0 (2.40–6.77)	2.2 (1.03–4.55)	0.042*
Living with domestic animal					
No	82	16	1	1	
Yes	122	80	3.4 (1.84–6.17)	3.4 (1.35–8.76)	0.009*
Habit of walking barefoot					
Yes	140	22	1	1	
No	64	74	7.4 (4.20–12.88)	4.3 (2.17–8.47)	0.000*
Not habit of hand washing before meal					
Yes	178	64	1	1	
No	26	32	3.4 (1.89–6.18)	3.4 (1.14–10.11)	0.028*
Residence					
Urban	97	26	1	1	
Rural	107	70	2.2 (1.28–3.62)	0.3 (0.05–2.48)	. 0.290
Age in years					
15–18	10	10	1	1	0.342
19–25	103	103	1.9 (0.40–9.25)	1.6 (0.20–12.88)	0.650
> 25	91	91	2.9 (0.62–14.05)	2.9 (0.32–25.60)	0.347
First pregnancy					
Yes	95	32	1	1	
No	109	64	1.7 (1.05–2.89)	2.1 (0.91–4.96)	0.080
Month of current pregnancy					
≤ 3	65	19	1	1	0.442
4–6	79	44	1.90 (1.01–3.57)	1.4 (0.57–3.26)	0.480
≥ 7	60	33	1.9 (0.97–3.66)	0.8 (0.33–2.22)	0.738
Have seen open defecation					
No	111	30	1	1	
Yes	93	66	2.6 (1.57–4.38)	1.2 (0.52–2.68)	0.687
Habit of eating raw fruit					
No	63	18	1	1	
Yes	141	78	1.7 (1.00–3.06)	1.9 (0.94–4.04)	0.071
Habit of bathing					
Yes	162	57	1	1	
No	42	39	2.6 (1.55–4.48)	0.7 (0.25–1.86)	0.452
Drinking water source					
Pipe	86	25	1	1	0.155
River	6	14	8.0 (2.79–23.05)	1.7 (0.25–11.10)	0.590
Well	69	36	1.8 (0.98–3.27)	0.5 (0.12–2.41)	0.407
Spring	43	21	8.0 (2.79–23.05)	0.4 (0.07–1.94)	0.234

*significant with ***p*** **< 0.05**

## Discussion

This study showed that the prevalence of hookworm infection was 32.0%. This finding is lower than the study conducted among pregnant women in Vietnam 78.15% [10], Andhra Pradesh 78.14% [11], Ghana, 45% [12] and Uganda 45% [13]. This low prevalence in this study might be geographic difference and time gap where those studies were conducted averagely 5 years ago. The finding of this study almost similar with the study conducted in Nigeria 35.8% [4]. The finding of this study had also higher prevalence of hookworm infection than the study done in Hossana, Ethiopia 7.0% [7], Addis Ababa, Ethiopia 1.3% [14] and Bahir dare, Ethiopia 5.5% [15]. This higher prevalence in this study might be due to socio demographic difference. In the previous studies the majority of participants were in urban dweller but in this study majority of participants were rural dweller. Pregnant women who lived in a single room were 2.8 times more likely to be infected by hookworm than pregnant women who lived in ≥2 living rooms. This finding is consistent to the study conducted in Southern Thailand [16]. This might be due to poverty stricken households with poor sanitation [1]. In this study, pregnant women whose monthly income less than or equal to 1500 Ethiopian birr were 3.7 times more likely to be infected by hookworm than pregnant women whose monthly family income greater than 1500 Ethiopian birr. This study is almost similar with the study done in Hossanna, Ethiopia [7]. This might to be due to not affording to buy shoes and might led to walking on barefoot which predisposes for hookworm infection [17]. Pregnant women who lived in household that have not toilet facility had almost 2.2 times more likely to be infected by hookworm than pregnant women who lived in households that have toilet facility. This finding is almost similar with the study conducted in southern Thailand [14]. This might be due to open defecation that lead contamination with faces may cause hookworm infection [18]. In this study, pregnant women with habit of barefoot had almost 4.3 times more likely to be infected by hookworm than those who did not the habit. This study is almost comparable with the study done in Thailand [16, 19], Dhare, India [20] and Hossana, Ethiopia [7]. Walking on barefoot might predispose to hookworm infection whose infective stage is found in soil [8, 17, 18, 21, 22]. The result of the study showed that pregnant women who had not habit of hand washing were 3.4 times more likely to be infected by hookworm than those who had habit of hand washing before meal. This study is almost similar with the study conducted in Peninsular Malaysia [23]. This might be due to contamination of hand with soil and faces which leads hookworm transmission through the ingestion of larvae [17].

In this study, pregnant women who lived with domestic animals had almost 3.4 times more likely infected by hookworm than those who did not live with domestic animals. This result is almost similar with the study conducted in Malaysia [23]. This might to be due to domestic animals may contaminate with human faces which contain hookworm larvae. It can lead a hookworm infection by touching contaminated animal [18]. The limitations of the study are less sensitive with compared to concentration diagnostic technique to detect light hookworm infection and the study also did not show the effect and the load of hookworm infection among pregnant women.

## Conclusion

This study showed that high prevalence of hookworm infection among pregnant women in the study area. Infections with hookworm among pregnant women were positively associated with not habit of hand washing before meal, living with a single room, monthly income, unavailability of latrine, living with domestic animals and habit of walking on barefoot. Therefore, Public health measures should contain to emphasis the importance of environmental, personal hygiene and preventive chemotherapy (deworming) is recommended as a public health intervention for all pregnant women in the study area as the base line prevalence of hookworm infections is high.

## Supplementary information

**Additional file 1.** Questionnaire (Amharic and English language version)

## Data Availability

The data sets generated during the current study are available from corresponding author on reasonable request.

## Abbreviations

ANC: Antenatal care
AOR: Adjusted odds ratio
CI: Confidence interval
COR: Crude odds ratio
SPSS: Statistical package for social science

## References

[1] Walana W, Nana E, Crowther S (2014). Prevalence of hookworm infection: a retrospective study in Kumasi. Asian Pac J Trop Biomed.

[2] Brooker S, Hotez PJ, Bundy DAP (2008). Hookworm-related Anaemia among pregnant women: a systematic review. PLoS Negl Trop Dis.

[3] Peter J. The development impact of the neglected tropical diseases (NTDs). Expert Paper. 2011:6–13.

[4] Alli JA, Okonko IO, Kolade AF, Nwanze JC, Dada VK, Ogundele M (2011). Prevalence of intestinal nematode infection among pregnant women attending antenatal clinic at the university college hospital, Ibadan, Nigeria. Pelagia Res Library.

[5] Baido SE, Tay SCK, Obiri-Danso K, Abruquah HH (2010). Intestinal helminth infection and anaemia during pregnancy: a community based study in Ghana. J Bacteriol Res.

[6] Bartsch SM, Hotez PJ, Asti L, Zapf KM, Bottazzi ME, Diemert DJ (2016). The global economic and health burden of human hookworm infection. PLoS Negl Trop Dis.

[7] Tesfaye D.J., Beshir W.G., Dejene T. and Tewelde, T. Prevalence of intestinal helminthiases and associated factors among pregnant women attending antenatal clinic of Nigist Eleni Mohammed Memorial Hospital, Hossana, Southern Ethiopia. Open Access Library J; 2015, 2: e1660. http://dx.doi.org/10.4236/oalib.1101660.

[8] Plotkin S, Diemert DJ, Bethony JM, Hotez PJ (2007). Hookworm vaccines. Oxf J Med Health Clin Infect Dis.

[9] Ethiopian fiscal year 2009 Dembecha health office annual plan, 2008, Dembecha.

[10] Sant R. P., Sonia R. C., Tran Q. P., Gerard J. C., Damien J., Sally K., Nong T. T.,Lachlan M.G., Antonio M. and Beverley A .B. Anemia, Iron deficiency, meat consumption, and hookworm infection in women of reproductive age in Northwest Vietnam. Am Soc Trop Med Hyg; 2008, 78(3): 375–381. PMC5619638 18337329

[11] Kudaravalli J, Madhavi S, Nagaveni D, Deshpande N, Rama M (2011). Anemia, Iron deficiency, meat consumption, and hookworm infection in women of reproductive age in rural area in Andhra Pradesh. Ann Biol Res.

[12] Humphries D, Mosites E, Otchere J, Twum WA, Woo L, Jones-Sanpei H, Harrison LM, Bungiro RD, Benham-Pyle B, Bimi L, Edoh D. Epidemiology of hookworm infection in Kintampo North Municipality, Ghana: patterns of malaria coinfection, anemia, and albendazole treatment failure. Am Soc Trop Med Hyg. 2011;84(5):792–800. 10.4269/ajtmh.2011.11-0003 PMC3083749 21540391

[13] Mpairwe H, Ndibazza J, Webb EL, Nampijja M, Muhangi L, Apule B, Lule S, Akurut H, Kizito D, Kakande M, Jones FM, Fitzsimmons CM, Muwanga M, Rodrigues LC, Dunne DW, Elliott AM (2014). Maternal hookworm modifies risk factors for childhood eczema: results from a birth cohort in Uganda. Pediatr Allergy Immunol.

[14] Intestinal GW (2015). Parasitic infection in pregnant women attending antenatal care at Gandhi Memorial Hospital, Addis Ababa, Ethiopia. Harar Bull Health Sci.

[15] Derso A, Nibret E, Munshea A (2016). Prevalence of intestinal parasitic infections and associated risk factors among pregnant women attending antenatal care center at Felege Hiwot referral hospital, Northwest Ethiopia. BMC Infect Dis.

[16] Liabsuetrakul T (2009). Epidemiology and the effect of treatment of soil-transmitted helminthiasis in pregnant women in southern Thailand. Southeast Asian J Trop Med Public Health.

[17] Hotez PJ, Bethony J, Bottazzi ME, Brooker S, Diemert D, Loukas A. New technologies for the control of human hookworm infection. Trends in parasitology. 2006;22(7):327–31. 10.1016/j.pt.2006.05.004 16709466

[18] Fiester RF. Division of Infectious Disease Department of Medicine University of Illinois Hospital PO Box 6990 Chicago, 111. 60616. Science. 1969;166:1032–4.

[19] Jiraanankul V, Aphijirawat W, Mungthin M, Khositnithikul R, Rangsin R, Traub RJ, Piyaraj P, Naaglor T, Taamasri P, Leelayoova S (2011). Incidence and risk factors of hookworm infection in a rural Community of Central Thailand. Am Soc Trop Med Hyg.

[20] Prajapati R, Bhagore J (2015). Study of intestinal parasite of adjacent villages of Dhar. Int J Sci Res (IJSR).

[21] Hotez PJ, Bethony J, Bottazzi ME, Brooker S, Buss P (2005). Hookworm: “The great infection of mankind.”. PLoS Med.

[22] Fenwick A. "The global burden of neglected tropical diseases”. Public Health; 2012, 126 (3): 233–236.Doi:10.1016/j. puhe. 2011.11.015. PMID 22325616. 10.1016/j.puhe.2011.11.015 22325616

[23] Ngui R, Aziz S, Chua KH, Aidil RM, Lee SC, Tan TK, Sani MM, Arine AF, Rohela M, Lim A (2015). Patterns and Risk Factors of Soil-Transmitted Helminthiasis among Orang Asli Subgroups in Peninsular Malaysia. Am Soc Trop Med Hyg.

