# Supplementary material for: Prevalence of hookworm infection and associated factors among pregnant women attending antenatal care at governmental health centers in DEMBECHA district, north West Ethiopia, 2017
Source: BMC Pregnancy Childbirth. 2020 Aug 12;20:457. doi: 10.1186/s12884-020-03134-0 (PMC7424972; doi:10.1186/s12884-020-03134-0)
Supplement: Supplementary file 1 — Additional file 1. Questionnaire (Amharic and English language version) [file 12884_2020_3134_MOESM1_ESM.docx]

# APPENDIX A

**Questionnaire**

**Consent Sheet**

**Code No__________**

Debre Markos university health science college department of public health

Questionnaire for Prevalence of hookworm infection and associated factors among pregnant women who attend ANC at governmental health centers in Dembecha district, North West, Ethiopia

Good morning/after noon

My name is-------------------------------- I am working as data collector in this study that assesses the Prevalence of hookworm infection and associated factors among pregnant women who attend ANC at governmental health centers in Dembecha district for an investigator doing her thesis for the partial fulfillment of Master’s degree in Reproductive health at Debre Markos university, Ethiopia. You are selected by chance. Participation in this survey is voluntary and you can choose not to take part. However, your willingness to answer all of the questions and giving stool sample would very important to you and other pregnant women. Your name will not be written in this form and the information you will give to us is kept confidential. There will be no injection, drawing of blood or any blood fluid involved. You will be interviewed and will give stool sample. It does not take more than 20 minutes. If the result of the stool examination positive for hookworm and other parasites, I will refer you to ANC clinic for medication. Generally, all information you will give will be used for only research purpose.

Are you voluntary to participate in this study?

A / yes B/ no

If the answer is yes, Thanks! Conduct

If the answer is no, Thanks! Transfer to the next respondent

Name and Signature of the data collector ______________

Date of interview_________

Name and signature of the supervisor ____________________Date_________

1. **Socio-demographic characteristics of study participants**

| S. No. | Question | Answer and Code | Skip |
| --- | --- | --- | --- |
| 101 | Where is your residence? | 1. .Urban 2. .Rural |  |
| 102 | Age? | ___________________________years |  |
| 103 | Marital status? | 1. Married 2. Single 3. Divorced 4. Widow |  |
| 104 | What is your level of education? | 1. Unable to read and write 2. Read and write but have no formal education 3. Primary 4. Secondary 5. Above secondary |  |
| 105 | What is your religion? | 1. Orthodox 2. Muslim 3. Catholic 4. Protestant 5. Others (specify)------------------------ |  |
| 106 | What is your occupation? | 1. House wife 2. Government employee 3. Farmer 4. Merchant 5. Others (specify)----------------------- |  |
| 107 | How much is your house hold average monthly income in birr; | ------------------------- |  |

**II. Environmental and housing factors**

| S.NO | Questions | code and Answer | Skip |
| --- | --- | --- | --- |
| 201 | Number of living rooms in the house | ------------------------ |  |
| 202 | Do you have latrine? | 1. Yes 2. No | If no skip to Q 205 |
| 203 | If yes for Q202, what types of latrine? | 1. Improved 2. Traditional |  |
| 204 | If yes for Q 202; How do use it? | 1. Always 2. Sometimes |  |
| 205 | If no to Q 202; Where you used? | 1. Open field 2. From neighbor toilet 3. Other (Specify it) |  |
| 206 | Have you seen open defecation in your locality? | 1. Yes 2. No | If no skip to Q208 |
| 207 | If yes to Q206; where? | 1. near the river 2. In the main road 3. Through the bush 4. other (specify)------------- |  |
| 208 | Is there any domestic animals living with you? | 1. Yes 2. No | If no skip to Q210 |
| 209 | If yes to Q208; which ones? | 1. Cat 2. Dog 3. Cow/ox 4. Goat 5. Sheep 6. other (specify)----------- |  |
| 210 | What is your source of drinking water? | 1. Pipe 2. River 3. Well 4. Spring 5. Others (specify)-------------- |  |
| 211 | If it is pipe water; Where is the site? | 1. From home 2. From public |  |
| 212 | If the answer of Q210 is well, what type of? | 1. Protected 2. Unprotected |  |
| 213 | If the answer of Q210 is spring, what type of? | 1. Protected 2. Unprotected |  |

**III. Personal factors**

| S.No | Questions | Answer and code | Skip |
| --- | --- | --- | --- |
| 301 | Do you have a habit of hand washing before meal? | 1. Yes 2. No | If no skip Q303 |
| 302 | If you say yes for Q301, how often? | 1. Always 2. Sometimes |  |
| 303 | Do you have a habit of taking bath? | 1. Yes 2. No | If no skip Q307 |
| 304 | If you say yes for Q303, how often? | 1. More than once a week 2. Once a week 3. Once a month 4. Others (specify)--------- |  |
| 305 | If you say yes for Q303, where do you take? | 1. Inside the house 2. Outside the house |  |
| 306 | If it is outside in Q 305, where? | 1. Pond 2. at water fall 3. others (specify) ----- |  |
| 307 | Do you have habit of eating raw fruit and vegetable? | 1. Yes 2. No | If No go to Q309 |
| 308 | If the answer in question 307 is yes, do you washing it before eating? | 1. Yes 2. No |  |
| 309 | Do you have habit of shoe wearing? | 1. Yes 2. No | If No go to Q311 |
| 310 | If yes for Q.309 how do wear it? | 1. Always 2. If I go to market 3. If there is ceremony 4. Others |  |
| 311 | If not for Q.309 What is your reason not wearing shoes? | 1. I cannot afford to buy 2. it is not comfortable to me 3. people laugh me while I am wearing shoe 4. any another (specify)------- |  |

**IV. Pregnancy related factors**

| S. No | Questions | Answer and code | Skip |
| --- | --- | --- | --- |
| 401 | Is this your first pregnancy? | 1. Yes 2. No | If yes for question 401,skip to Q403 |
| 402 | If not for Q. 401 How many times you become pregnant including the present one? | ---------------------- |  |
| 403 | What is the gestational age of this pregnancy in month? | --------------------- |  |

#

# APPENDIX B

**Laboratory result report format**

Direct saline preparation

Hookworm/presence or absence

1. Ova /parasite seen
2. No ova/parasite seen

**Standard operating procedure (SOP) for hookworm**

**DIRECT SMEAR EXAMINATION**

First the stool sample was examined macroscopically (Noted for color, consistency and look for mucus, blood stains and worms).

A glass slide with the patient name/ code was leveled.

Only fresh specimens and refuse specimens contaminated dirty or urine was accepted.

One drop of normal saline in the middle of the left half of the slide was put, and then small piece of stool was taken with wooden applicator.

If the sample could not be examined immediately, it was left in a cool place and not exposed to sun.

If the stool was formed take the piece from inside and the surface of the sample.

If the stool is liquid take a drop. Any part is o.k.

If the specimen very liquid place one or two drop of stool directly on to the slide and cover it. With cover slide, did not add the saline as this would farther dilute the specimen.

Mix the sample first with the drop of normal saline on the left half of the slide.

Place the cover slip over each drop.

Put the cover slip slowly

Letting it move down from the slide to avoid air bubble.

Use 10x and 40x objectives.

Examine the entire cover slip systematically.

# APPENDIX C

**Materials and Laboratory Procedure**

**Materials**

- Microscope
- Stool cup
- Slide
- Cover slide
- 10% normal saline
- Glove
- pen, marker, pencil
- Gauze
- questionnaire paper
- Applicator stick

**Laboratory Procedure**

After all participants interviewed, they requested to give stool sample in clean dry cup. Similar codes were written on the cups with questionnaires and the laboratory request formats.

A drop of fresh physiological saline was placed on a clean slide approximately 1 gram of stool sample was added. The preparation was covered with cover slip and a fecal sample of all study participants were examined under microscope using 10× and 40× objectives respectively with the condenser iris closed sufficiently to give good contrast for the presence or absence of hookworm ova/parasite.

**መጠይቅ**

**የኮድ ቁጥር**

**የስምምነት ቅጽ**

ደብረ ማርቆስ ዩኒቨርስቲ ጤና ሳይንስ ኮሌጅ የህብረተሰብ ጤና ትምህርት ክፍል

በደንበጫ ወረዳ በሚገኙ ስድስት ጤና አጠባበቅ ጣቢያዎች የነፍሰጡር ክትትል የሚያደርጉ ነፍሰጡር እናቶች ላይ የመንጠቆ ትል መጠን እና እና ከዚህ ጋር ግንኙነት ያላቸውን ነገሮች ለማጥናት የተዘጋጀ ጥናታዊ ጽሁፍ መጥይቅ

ጤና ይስጥልኝ -------------እባላለሁ፡፡ በደንበጫ ወረዳ ክትትል የሚያደርጉ ነፍሰጡር እናቶች ላይ የመንጠቆ ትል መጠን እና ከዚህ ጋር ግንኙነት ያላቸውን ለማጥናት በተዘጋጀው ጥናታዊ ጽሁፍ መረጃ በመሰብሰብ የምሳተፍ አንዱ ነኝ፡፡ ይህንን ጥናታዊ ጽሁፍ የምታጠናው በደብረ ማርቆስ ዩኒቨርስቲ በጤና ሳይንስ ኮሌጅ በህብረተሰብ ጤና ትምህርት ክፍል የስነ - ተዋልዶ ጤና ድህረ- ምረቃ ተመራቂ ናት፡፡እርሰዎ የተመረጡት በአጋጣሚ ነው፡፡ በዚህ ጥናታዊ ጽሁፍ ላይ መሳተፍም አለመሳተፍም ይችላሉ፡፡ ነገር ግን ቢሳተፉ ለእርሰዎም ሆነ ለሌሎች በጣም ጠቃሚ ነው፡፡ በመጠየቁ ላይ ስመዎት አይፃፍም፡፡የሚሰጡት መረጃ ሚስጥሩ ይጠበቃል፡፡መርፌ መወጋትም ሆነ ደም መስጠት አይጠበቅበዎትም፡፡ የተወሰኑ ጥያቄዎች እጠይቀዎታለሁ እንዲሁም የሰገራ ናሙና ይሰጣሉ፡፡ መጠይቁም ሆነ የሰገራ ምርመራው ከ20 ደቂቃ በላይ አይወስድም፡፡ የሰገራ ምርመራ ውጤት የመንጠቆ ትል ከተገኘ የዚህን ህክምና ወይም መድሃኒት ወደሚያገኙበት ክፍል እልከዎታለሁ፡፡ ከእርሰዎ የተገኘው አጠቃላይ መረጃ የምንጠቀምበት ለጥናታዊ ጽሁፍ ብቻ ነው፡፡

በጥናቱ ላይ ለመሳተፍ ፍቃደኛ ነዎ;

ሀ/ አዎ----------------መልሱ አዎን ከሆነ ወደ ሚቀጥለው ጥያቄ እለፍፊ

ለ/ አይደለሁም---------መልሱ የለም ከሆነ አመስግነለሁ ብለህሽ ጥያቄውን አቋርጪጥ

መረጃ ሰብሳቢ

ስምፊርማቀን

ተቆጣጣሪ ስምፊርማቀን

**ክፍል፡− የማህበራዊ እና የስነ−ህዝብ ጥያቄዎች**

| **ተቁ** | **ጥያቄ** | **ምላሽ እና ኮድ** | **ይለፍ** |
| --- | --- | --- | --- |
| 101 | የመኖሪ ቦታ | 1.ገጠር  2. ከተማ |  |
| 102 | እድሜዎ ምን ያህል ነው; | ------በአመት |  |
| 103 | የጋብቻሁኔታ | 1. ባለትዳር 2. ያላገባች 3. የፈታች 4. የትዳር አጋβ የሞተባት |  |
| 104 | የትምህርት ደረጃ | 1. ማንበብ እና መፃፍ የማትችል 2. ማንበብ እና መፃፍ የምትችል 3. አንደኛ ደረጃከኛ ክፍል 4. ሁለተኛ ደረጃ ከ ኛ ክፍል 5. ከሁለተኛ ደረጃ በላይ |  |
| 105 | ሃይማኖት | 1. ኦርቶዶክስ 2. እስልምና 3. ካቶሊክ 4. ፕሮቴስታንት 5. ሌላ  ይገለጽ  |  |
| 106 | ስራ | 1. የቤት ዕመቤት 2. የመንግስት ሰራተኛ 3. ግብርና 4. ነጋዴ 5. ሌላይገለጽ |  |
| 107 | የቤተሰባችሁ የወር ገቢ በብር ስንት ነው; | -------- |  |

**2.አካባቢያዊ እና የመኖሪያ ቤቱን የሚመለከቱ ጥያቄዎች**

| **ተ.ቁ** | **ጥያቄ** | **ኮድ እና መልስ** | **ይለፍ** |
| --- | --- | --- | --- |
| 201 | የመኖሪያ ቤተዎ ስንት ክፍል ነው; | ------------ |  |
| 202 | መጸዳጃ ቤት አለዎት | 1.አዎ  2.የለም | መልስዎ የለም ከሆን ወደ ጥያቄ 205 እለፍ/እለፊ |
| 203 | የጥያቄ ቁጥር 201 መልስ አዎ ከሆነ የመፀዳጃ ቤት አይነቱ ምንድን ነው; | 1. የተለምዶ   2.የተሻሻለ |  |
| 204 | የጥያቄ 201 መልሰዎ አዎ ከሆነ እንዴት ይጠቀሙበታል; | 1.ሁልጊዜ  2.አልፎአልፎ |  |
| 205 | የጥያቄ ቁጥር 201 መልሰዎ የለም ከሆነ የት ይፀዳዳሉ; | 1. ሜዳ ላይ 2. ከጎረቤት መጸዳጃ ቤት 3. ሌላ ከሆነ ይገለጹ |  |
| 206 | እርሰዎ ከሚኖሩበት አካባቢ ሰዎች ሜዳ ላይ ሲፀዳዱ አይተው ያውቃሉ; | 1. አዎ   2.የለም | መልሰዎ የለም ከሆነ ወደ ጥያቄ ቁጥር 208 እለፍ/ እለፊ |
| 207 | የጥያቄ204 መልሰዎ አዎ ከሆነ የት ነው ሲፀዳዱ ያዩት; | 1. ወንዝ አካባቢ 2. ከዋናው መንገድ ላይ 3. ከቁጥቋጥ ውስጥ 4. ከዚህ ውጭ ከሆነ ይጥቀሱ |  |
| 208 | ከእርሰዎ ጋር የሚኖሩ የቤት እንስሳት አሉ; | 1. አዎ   2.የለም | መልሰዎ የለም ከሆነ ወደ ጥያቄ ቁጥር 210 እለፍ/ፊ |
| 209 | የጥያቄ 208 መልሰዎ አዎ ከሆነ የትኛው እንስሳት; | 1. ድመት 2. ውሻ 3. ላም/በሬ 4. ፍየል 5. በግ 6. ሌላ ካለ ይጠቀስ |  |
| 210 | የመጠጥ ውሃ ከየት ነው የሚያገኙ; | 1. ከቧንቧ 2. ከወንዝ 3. ከጉርጓድ 4. ከምንጭ 5. ሌላ ከሆነ ይጠቀስ |  |
| 211 | የጥያቄ ቁጥር210 መልሰዎ የቧንቧ ውሃ ከሆነ የት ነው; | 1.ከቤት  2.የህዝብ |  |
| 212 | የጥያቄ ቁጥር 210 መልሰዎ የጉድጓድ ከሆነ አይነቱ ምንድን ነው; | 1.የተጠበቀ  2.ያልተጠበቀ |  |
| 213 | የጥያቄ ቁጥር 210 መልሰዎ የምንጭ ከሆነ አይነቱ ምንድን ነው; | 1.የተጠበቀ  2.ያልተጠበቀ |  |

**3.ከግለሰቧ ጋር የተየያዙ ጥያቄዎች**

| **ተ.ቁ** | **ጥያቄ** | **ኮድ እና መልስ** | **ይለፍ** |
| --- | --- | --- | --- |
| 301 | ከመመገበዎ በፊት እጅዎን የመታጠብ ልምድ አለዎት; | 1.አዎ  2.የለም | መልሰዎ የለም ከሆነ ወደ ጥያቄ ቁጥር 303 እለፍ/ፊ |
| 302 | የጥያቄ ቁጥር 301 መልሰዎ አዎ ከሆነ ምን ያህል ጊዜ; | 1.ሁልጊዜ  2.አልፎ አልፎ |  |
| 303 | ገላዎን የመታጠብ ልምድ አለዎት; | 1.አዎ  2.የለም | መልሰዎ የለም ከሆነ ወደ ጥያቄ ቁጥር 307 እለፍ/ፊ |
| 304 | የጥያቄ ቁጥር 303 መልሰዎ አዎ ከሆነ ምን ያህል ጊዜ; | 1. በሳምንት ከአንድ ጊዜ በላይ 2. በሳምንት አንድ ጊዜ 3. በወር አንድ ጊዜ 4. ሌላ ከሆነ ይገለጽ |  |
| 305 | የጥያቄ ቁጥር 303 መልሰዎ አዎ ከሆነ የሚታጠቡ የት ነው; | 1.ከቤት ውስጥ  2.ከቤት ውጭ |  |
| 306 | የጥያቄ ቁጥር 305 ከቤት ውጭ ከሆነ የት; | 1. ኩሬ 2. ከፏፏቴ 3. ሌላ ከሆነ ይገለጽ |  |
| 307 | በእሳት ያልበሰለ አትክልት እና ፍራፍሬ የመመገብ ልምድ አለዎ; | 1. አዎ   2.የለም | መለሰዎ የለም ከሆነ ወደ ጥያቄ ቁጥር 309 እለፍ/ፊ |
| 308 | የጥያቄ ቁጥር 307 መልስ አዎ ከሆነ አጥበው ነው የሚመገቡ; | 1.አዎ  2.የለም |  |
| 309 | ጫማ የማጥለቅ/መልበስ ልምድ አለዎ; | 1.አዎ  2.የለም | መልሰዎ የለም ከሆነ ወደ ጥያቄ ቁጥር 311 እለፍ/ፊ |
| 310 | የጥያቄ ቁጥር መልሰዎ አዎ ከሆነ እንዴት ነው የሚጠቀሙት; | 1. ሁልጊዜ 2. ወደ ገቢያ በመሄድበት ጊዜ 3. በባዓላት ቀን 4. ላሌ ከሆነ ይጠቀስ |  |
| 311 | የጥያቄ ቁጥር መልሰዎ የለም ከሆነ ጫማ የማያጠልቁበት ምክንያት ምንድን ነው; | 1. የመግዛት አቅም ስሌለኝ 2. ምቾት ስለማይሰጠኝ 3. ሰው ስለሚስቅብኝ 4. ሌላ ከሆነ ይጠቀስ |  |

1. **ከእርግዝና ጋር የተየያዙ ጥያቄዎች**

| **ተ.ቁ** | **ጥያቄ** | **ኮድ እና መልስ** | **ይለፍ** |
| --- | --- | --- | --- |
| 401 | ይህ እርግዝና የመጀመሪያዎ ነው; | 1.አዎ  2.አይደለም | መልሰዎ አዎ ከሆነ ወደ ጥያቄ ቁጥር 403 እለፍ/ፊ |
| 402 | የጥያቄ ቁጥር 401 መልስ አይደለም ከሆነ ስንተኛ እርግዝናዎ ነው; | ------ |  |
| 403 | የስንት ወር ነፍሰጡር ነዎ; | --------- |  |

**የሰገራ ምርመራ ውጤት**

1.የመንጠቆ ትል አለ

2.የመጠቆ ትል የለም
